# Photon-Counting Detector CT Enables Quantification of Dorsal Root Ganglion Enlargement in Radiculopathy

**DOI:** 10.3390/diagnostics16213461

**Published:** 2026-10-23

**Authors:** Simon Weiner, Sarah Perleth, Silvia Herbold, Anna Ryskal, Yanru Wang, Xinyu Zhang, Florian Hessenauer, Heike L. Rittner, Mirko Pham, Magnus Schindehütte

**Affiliations:** 1 Department of Diagnostic and Interventional Neuroradiology, University Hospital Würzburg, 97080 Würzburg, Germany; 2 Department of Neurosurgery, University Hospital Würzburg, 97080 Würzburg, Germany; 3 Department of Anaesthesiology, Intensive Care, Emergency and Pain Medicine, Centre for Interdisciplinary Pain Medicine, University Hospital Würzburg, 97080 Würzburg, Germany

**Keywords:** dorsal root ganglion, radiculopathy, periradicular therapy, photon-counting detector computed tomography

## Abstract

**Background/Objectives**: Dorsal root ganglion (DRG) enlargement in radiculopathy reflects inflammatory nerve root responses and correlates with symptom severity. While DRG enlargement can be detected by MRI, it is unclear whether photon-counting detector CT (PCD-CT) acquired during routine periradicular therapy (PRT) enables detection and quantification of DRG volumetric changes in lumbosacral radiculopathy. **Methods**: This retrospective study included 28 selected patients with unilateral L5 or S1 radiculopathy undergoing PCD-CT-guided PRT followed by confirmed therapeutic response. Three independent blinded readers segmented DRGs on low-dose planning acquisitions. Affected and contralateral DRG volumes were compared using paired Wilcoxon signed-rank tests. **Results**: Affected DRGs exceeded contralateral volumes in 26 of 28 patients (92.9%). Median symptomatic DRG volume was 341.1 mm^3^ versus 282.3 mm^3^ contralaterally (*p* < 0.001). Median DRG enlargement was +24.0%. The result was consistent across L5 and S1 radiculopathy levels. Inter-reader agreements were high (median Dice similarity coefficients 0.78–0.85, median volumetric similarities 0.93–0.95). **Conclusions**: PCD-CT enables visualization and quantification of DRG volumetric changes on routine therapeutic imaging of selected patients, extracting biological information from procedural acquisitions. This establishes PCD-CT for investigating nerve pathophysiology and extends CT-based neuroradiological research into biological questions previously limited to MRI.

## 1. Introduction

Lumbosacral radiculopathy causes significant morbidity through nerve root compression, typically manifesting as radiating leg pain and sensory-motor deficits [1,2]. The dorsal root ganglion (DRG) harbors sensory neuron cell bodies and undergoes inflammatory swelling in symptomatic radiculopathy [3,4]. This volume enlargement can be detected by MRI and correlates with symptom severity and treatment response [5,6]. However, MRI-based DRG assessment requires dedicated imaging sessions separate from therapeutic procedures.

CT-guided periradicular infiltration therapy (PRT) is an established intervention that delivers targeted anti-inflammatory medication to compressed nerve roots [7]. PRT routinely generates cross-sectional imaging data for procedural guidance. Conventional CT lacks sufficient soft tissue resolution to visualize DRG morphology at diagnostically acceptable radiation doses, rendering these periinterventional datasets purely procedural. If DRG pathology could be reliably assessed on routine low-dose procedural CT, morphological evaluation would become feasible without additional examinations, radiation exposure, or MRI requirements.

Photon-counting detector CT (PCD-CT) directly counts individual X-ray photons while measuring their energies, eliminating electronic noise, and enabling smaller detector elements without dose penalty [8]. This architecture delivers substantially higher spatial resolution and contrast-to-noise ratio than conventional energy-integrating detector CT. Clinical neuroradiological applications have demonstrated enhanced visualization of intracranial vascular devices [9] and embolic materials [10], superior temporal bone microstructure depiction [11,12], and sharper spinal anatomy delineation at reduced radiation doses [13]. Soft tissue structures like DRGs that are inadequately visualized by conventional CT might now be assessable with PCD-CT technology applied to routine low-dose protocols.

We investigated whether periinterventional PCD-CT images acquired during standard-of-care low-dose PRT provide sufficient quality for reliable DRG volumetry in selected patients with L5 or S1 radiculopathy. We tested two hypotheses: first, whether lumbar DRGs at the L5 and S1 levels can be consistently segmented on periinterventional PCD-CT datasets by multiple independent raters with high interrater agreement; second, whether volume differences between symptomatic and asymptomatic contralateral DRGs are detectable in patients with confirmed single-level, unilateral radiculopathy. Documented therapeutic response to PRT at the L5 or S1 level functionally confirmed the affected nerve root in each patient. This exploratory study aims to establish the methodological foundation for CT-based DRG morphometry and to explore its potential to augment the diagnostic evaluation of radicular pain syndromes.

## 2. Materials and Methods

### 2.1. Study Design and Patient Selection

**Figure 1:**
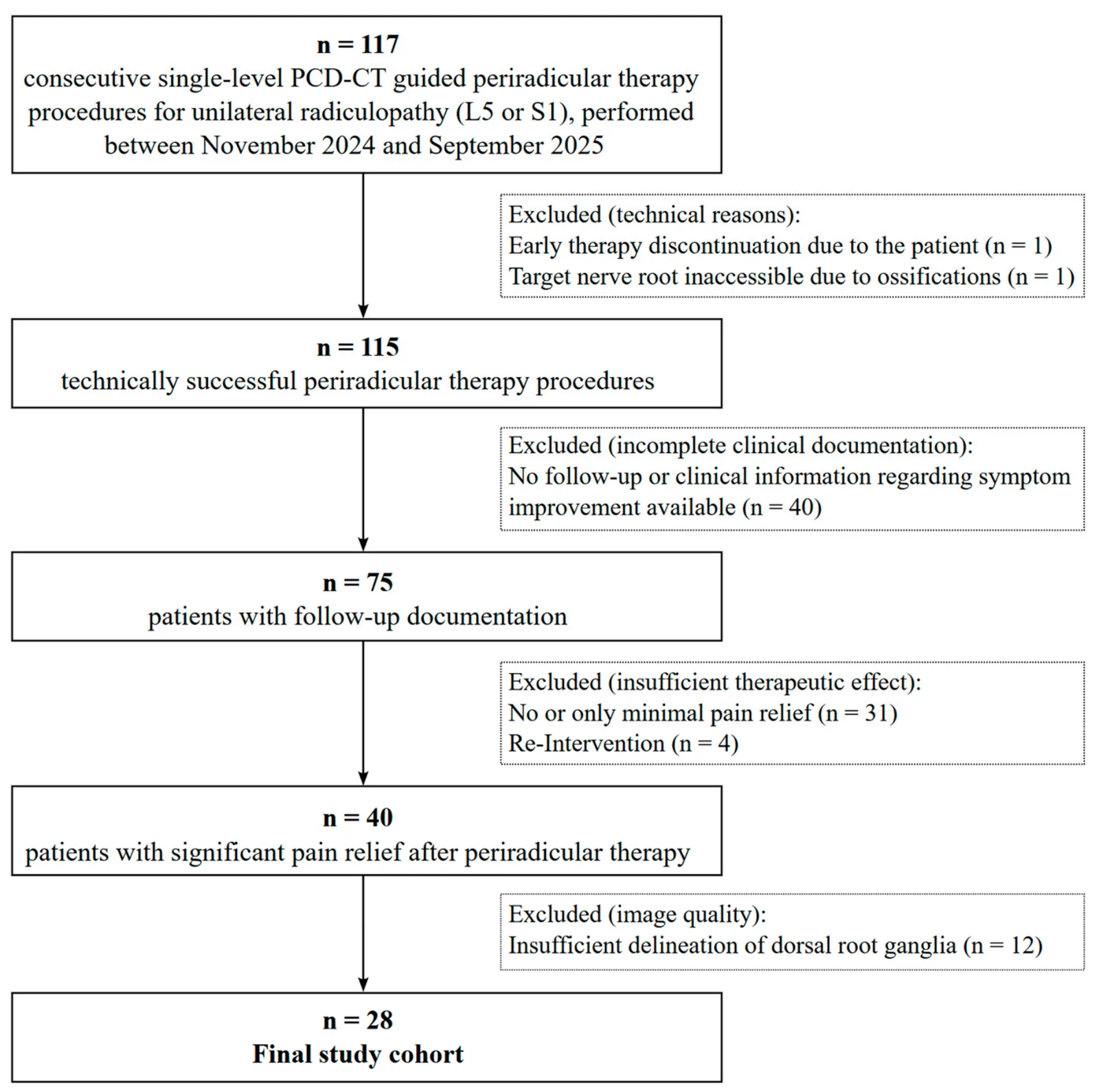
Selection of the study cohort.

This single-center retrospective exploratory study investigated DRG morphology using periinterventional PCD-CT datasets. The primary objective was to identify patients with single-level, unilateral radiculopathy at the L5 or S1 level, confirmed by documented therapeutic response to PRT. All consecutive patients who underwent unilateral single-level PCD-CT-guided PRT at the L5 or S1 level between November 2024 and September 2025 were initially screened (*n* = 117). The patient selection process is summarized in Figure 1.

Inclusion criteria comprised: (i) age ≥18 years, (ii) unilateral single-level radiculopathy at L5 or S1 level, (iii) PCD-CT-guided single-level PRT performed during the study period, (iv) technically successful PRT, (v) availability of clinical follow-up documentation, (vi) documented clinical improvement following PRT, defined as a marked reduction in radicular pain symptoms at follow-up examination as assessed by the treating physician, and (vii) sufficient image quality with adequate delineation of the DRG.

Exclusion criteria were: (i) technical failure of PRT, (ii) incomplete clinical follow-up data, (iii) insufficient therapeutic response, (iv) inadequate image quality precluding reliable DRG delineation, or (v) refusal to permit use of anonymized data for research purposes.

After applying inclusion and exclusion criteria, the final study cohort comprised 28 patients. Demographic, clinical, and imaging data were extracted in pseudonymized form for analysis. Baseline patient characteristics are shown in Table 1. Patients excluded for insufficient therapeutic response were segmented and analyzed post hoc using the same methodology; a description of this subcohort is provided in Table S1.

### 2.2. PCD-CT-Guided Periradicular Infiltration Therapy

All PRT procedures were performed under CT guidance using a first-generation clinical dual-source PCD-CT scanner (NAEOTOM Alpha.Prime; Siemens Healthineers, Erlangen, Germany; software version syngo CT VB20A).

Patients were positioned prone on the CT table. For trajectory planning, an initial dataset of the lumbar spine was acquired using an ultra-fast helical scan mode (i-Spiral) with the following parameters: tin-filtered 100 kV spectrum (0.4 mm tin prefiltration), automated exposure control (CARE Dose4D, Siemens Healthineers), matrix size of 512 × 512 pixels, gantry rotation time of 0.5 s, helical pitch factor of 0.8, single detector collimation width of 0.4 mm, and total detector collimation width of 57.6 mm. The field of view was 504 mm. Images were reconstructed with a high-resolution bone kernel (Br60f) at a slice thickness of 1.5 mm with contiguous slices (1.5 mm increment).

For intraprocedural needle positioning control, additional low-dose monitoring scans of the target level were obtained using a sequential acquisition scan mode (i-Sequence). Scan parameters were: tin-filtered 140 kV spectrum (0.4 mm tin prefiltration), nominal tube current-time product of 25 mAs (500 ms rotation time, 50 mA), total detector collimation width of 19.2 mm, matrix size of 512 × 512 pixels, and gantry rotation time of 0.5 s.

Radiation exposure during the procedure was low, as a low-dose technique is sufficient for trajectory planning. The dose–length product (DLP) for the complete intervention was 25.8 mGy × cm (range, 22.9–30.5 mGy × cm). For the planning spiral alone, the DLP was 14.3 mGy × cm (range, 12.3–18.8 mGy × cm), with a corresponding volumetric CT dose index (CTDIvol) of 1.6 mGy (range, 1.4–2.0 mGy).

**Figure 2:**
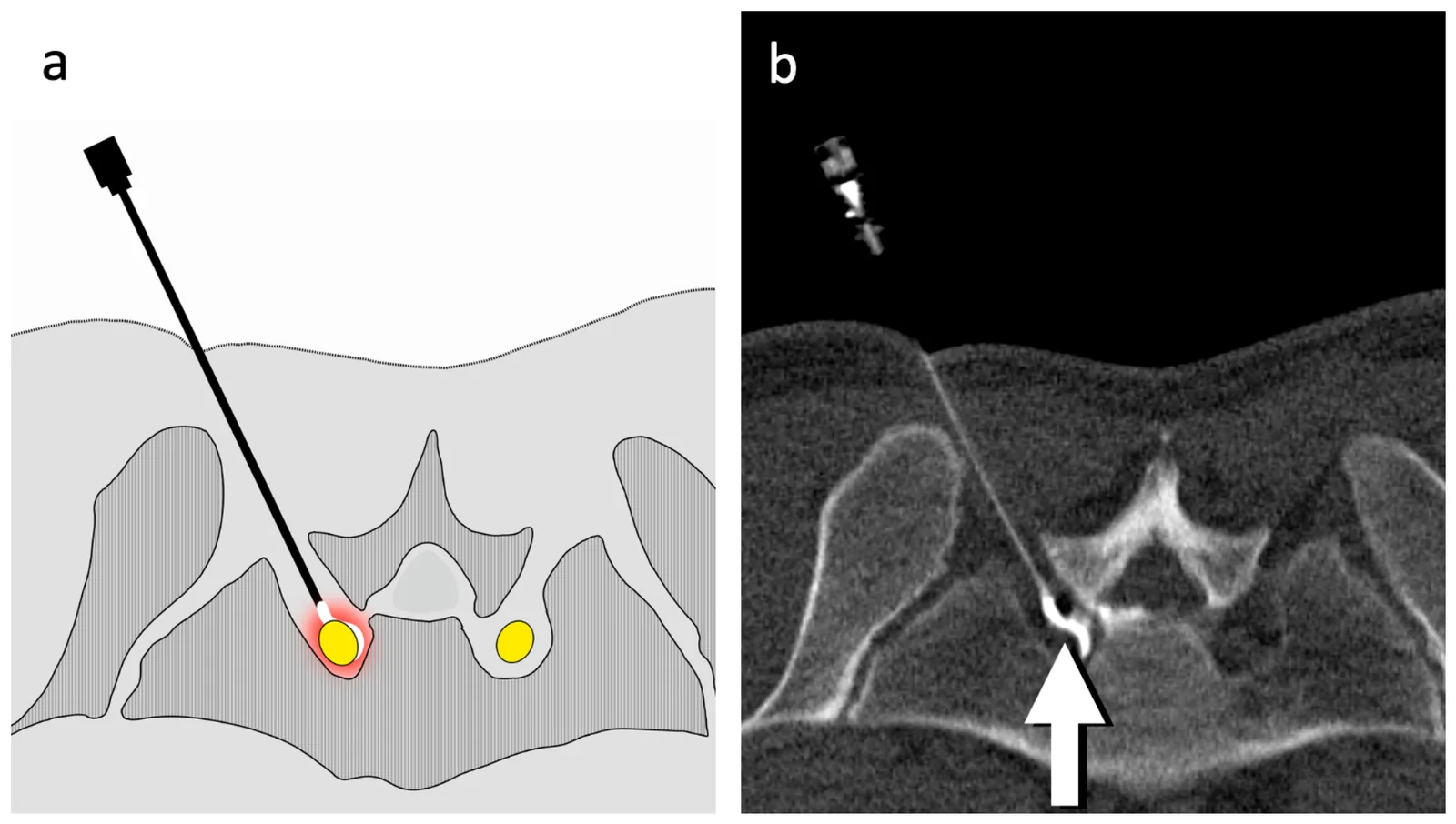
(**a**) Schematic illustration of a left S1 periradicular therapy and (**b**) normal periradicular contrast distribution on the axial control scan (white arrow).

Accurate needle placement was confirmed by visualizing appropriate periradicular contrast medium distribution (0.5 mL iopamidol, Solutrast 200M, Bracco Imaging Deutschland GmbH, Konstanz, Germany; Figure 2b). Following confirmation, periradicular injection of local anesthetic (1.5 mL bupivacaine hydrochloride, Bucain 0.5%, Puren Pharma GmbH & Co. KG, München, Germany) and a steroid (1.0 mL triamcinolone acetonide 40 mg/mL, TriamHEXAL 40, Hexal AG, Holzkirchen, Germany) was accomplished.

### 2.3. Image Acquisition Protocol for DRG Segmentation

For DRG morphological analysis, coronal multiplanar reformations were generated from the planning spiral acquisition using a dedicated post-processing workstation (syngo.via, version VB80G; Siemens Healthineers, Erlangen, Germany). The reconstruction parameters were a slice thickness of 1.5 mm with contiguous slices, a reconstructed matrix of 896 × 1142 pixels, and in-plane pixel spacing of 0.200 × 0.200 mm. A high-resolution bone kernel (Br56f) was applied. Images were displayed using a window setting of 450 Hounsfield units (HU) center and 1500 HU width.

### 2.4. Image Analysis and DRG Segmentation

Three readers with 2, 7, and 8 years of experience in neuroimaging independently performed three-dimensional segmentation of the DRGs using open-source software (3D Slicer, version 5.10.0; available at https://www.slicer.org/). Readers were blinded to the patients’ symptomatic side and to each other’s assessments. DRG boundaries were defined anatomically as the fusiform enlargement of the dorsal nerve root between the intervertebral foramen and the spinal cord. Segmentation was performed for bilateral DRGs of the same level on coronal reformations. A consensus ground-truth segmentation was automatically derived from the three independent manual segmentations using the Simultaneous Truth and Performance Level Estimation (STAPLE) algorithm with default parameters [14].

### 2.5. Outcome Measures

The primary outcome measures were (i) absolute DRG volumes at the L5 and S1 levels (in mm^3^) and (ii) within-patient volume ratio between symptomatic and asymptomatic contralateral DRGs. Secondary outcomes included spatial agreement metrics between independent raters (Dice similarity coefficient, DSC; volume similarity, VS) and exploratory associations between DRG volume and demographic variables (age, sex, height, weight, and body mass index).

### 2.6. Statistical Analysis

All statistical analyses and visualizations were performed using R (version 4.5.2; The R Foundation for Statistical Computing, Vienna, Austria; available at https://www.r-project.org/) and GraphPad Prism (version 10.2.3; GraphPad Software, San Diego, CA, USA; available at https://www.graphpad.com/).

Spatial agreement between the three independent DRG segmentations was quantified using the Dice similarity coefficient [15] and volume similarity [16], with values closer to 1 indicating better volumetric agreement.

Given the small sample size (*n* = 28) and exploratory nature of this investigation, exclusively non-parametric statistical tests were applied. Descriptive statistics are presented as median and interquartile range (IQR) for continuous variables or as number and percentage for categorical variables.

Within-patient comparisons of affected versus unaffected contralateral DRG volume were performed using the paired Wilcoxon signed-rank test. Statistical significance was defined as two-sided *p* < 0.05.

For each patient, a ratio between the volume of the affected DRG and its contralateral counterpart was calculated to determine the median percentage change across the cohort.

Associations between DRG volume and demographic variables (age, sex, height, weight, and body mass index) were assessed using Spearman’s rank correlation coefficient. These correlation analyses were exploratory and not adjusted for multiple testing.

### 2.7. Use of Generative AI

During the preparation of this manuscript, the authors used Claude Opus 5 (Anthropic, San Francisco, CA, USA) for language editing of author-written text, that is, to improve grammar, clarity, and readability. The tool was not used for the study design, for data collection, for image analysis or DRG segmentation, for the statistical analysis or the interpretation of the data, nor for the generation of any figures or tables. After using this tool, the authors reviewed and edited the text as needed and take full responsibility for the content of this publication.

## 3. Results

Twenty-eight patients (L5 radiculopathy: *n* = 19 [67.9%], S1 radiculopathy: *n* = 9 [32.1%]) met the inclusion criteria and were included in the final analysis (Table 1).

Pairwise comparison of the three readers’ segmentations demonstrated a median DSC ranging from 0.78 to 0.85 and a median VS ranging from 0.93 to 0.95. Detailed comparison metrics are shown in Table 2.

**Figure 3:**
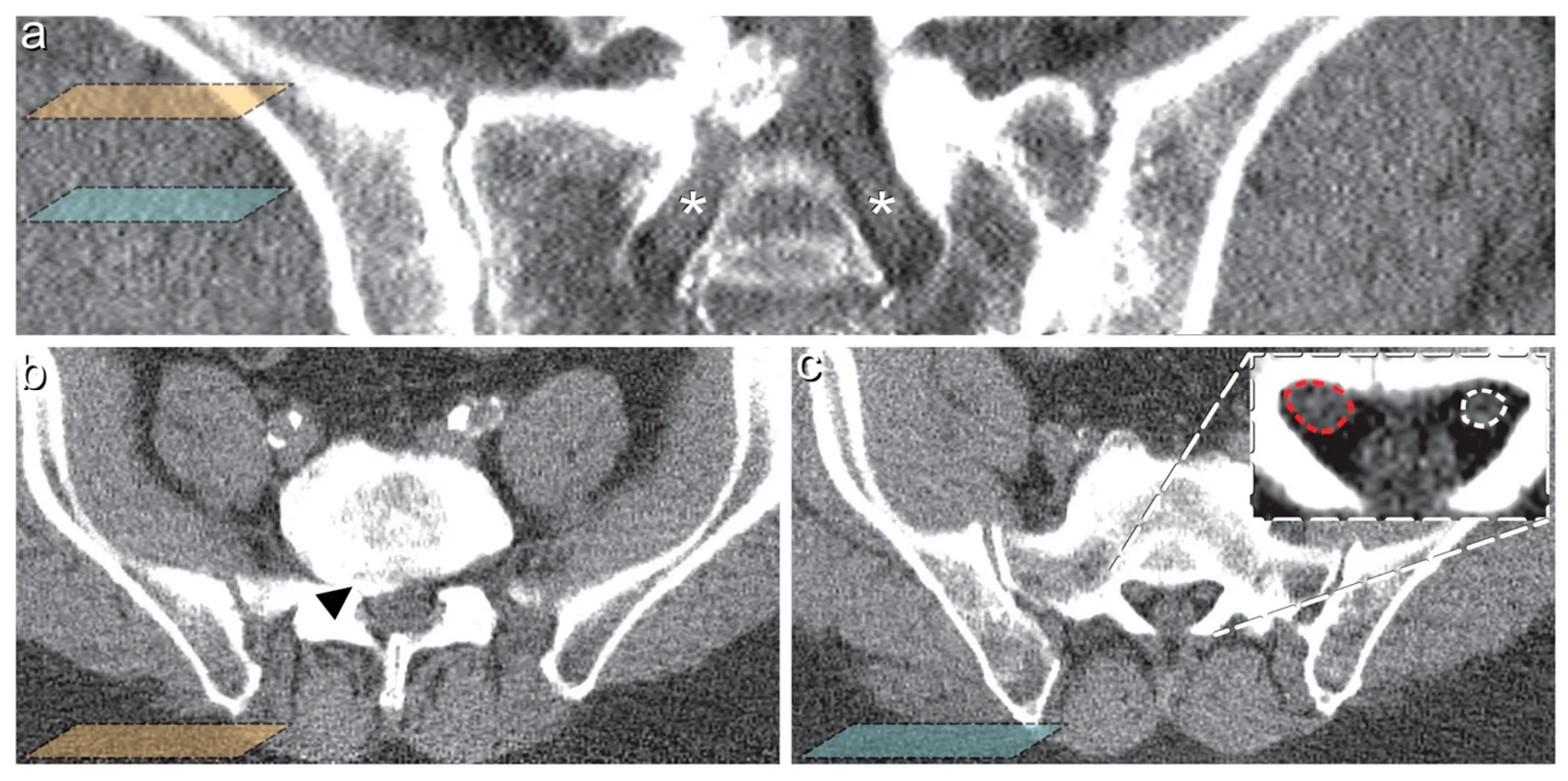
Representative case of bony lateral recess stenosis with right-sided dorsal root ganglion (DRG) compression. (**a**) Coronal multiplanar reformation provides an overview of the lumbar spine with bilateral DRGs at the affected level indicated by asterisks. (**b**) Axial section at the level of the lateral recess demonstrates bony recess stenosis with foraminal narrowing and mechanical compression of the right DRG (arrowhead). (**c**) Axial section at the level of the DRGs with corresponding magnified insets. Dotted-line contours delineate the segmented DRG boundaries bilaterally. The symptomatic right DRG appears markedly enlarged compared to the contralateral side, suggestive of inflammatory oedematous swelling, whereas the non-symptomatic left DRG is of normal size and morphology.

In 26 of 28 cases (92.9%), the affected DRG volume exceeded the contralateral unaffected DRG volume, based on the STAPLE-derived consensus segmentations. A representative case of DRG enlargement due to bony lateral recess stenosis is shown in Figure 3.

**Figure 4:**
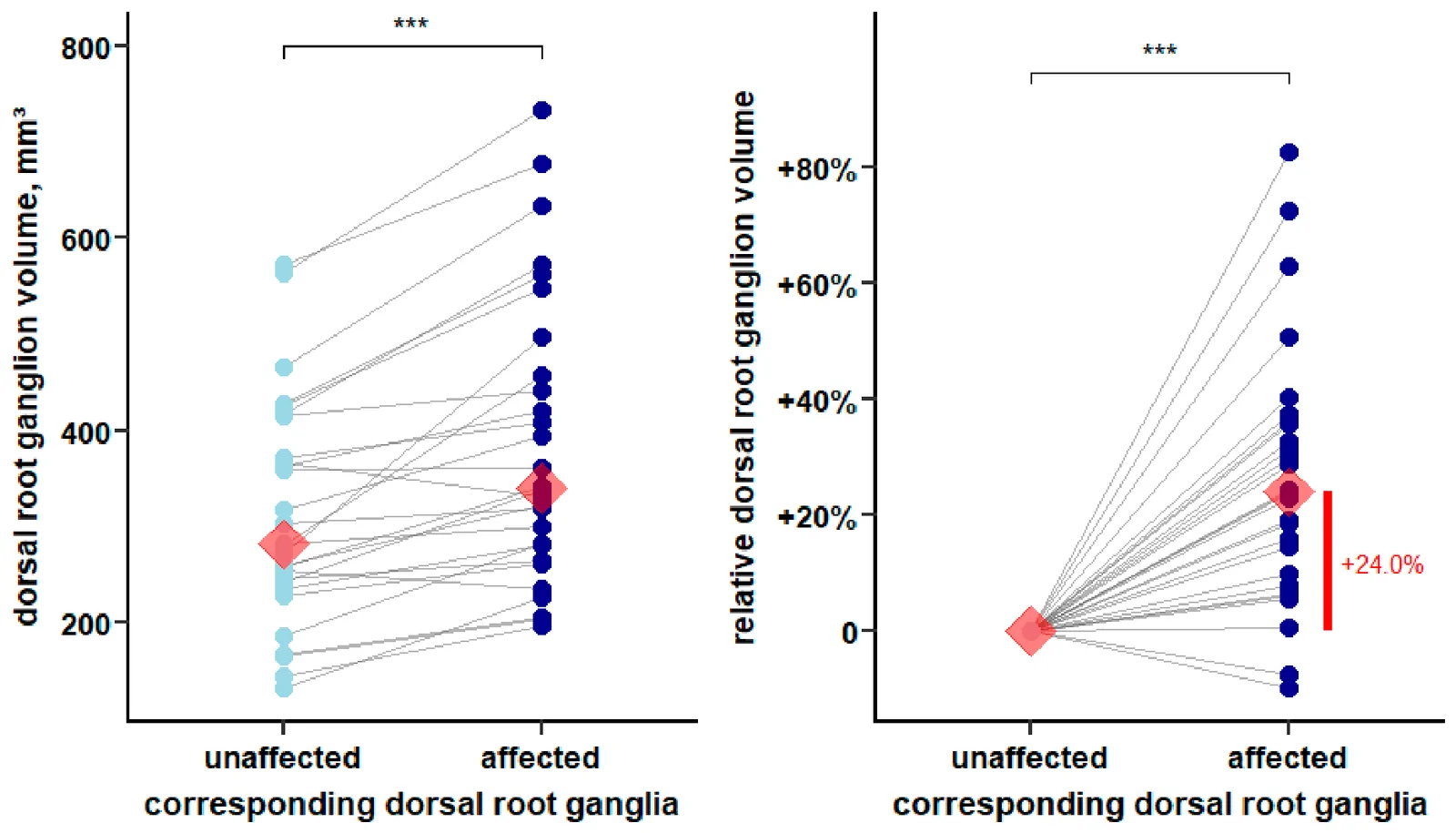
Paired comparison of DRG volumes between unaffected and affected sides. (**Left**) Absolute volumes per patient, with group medians indicated by red diamonds. (**Right**) Relative volumes expressed as percentage difference between affected and contralateral sides, demonstrating a median increase of +24.0% in affected dorsal root ganglia. Each pair of connected points represents one patient. Abbreviation: *** *p* < 0.001.

Median DRG volume on the symptomatic side was significantly enlarged to 341.1 mm^3^ (IQR, 277.6–468.0 mm^3^) compared with 282.3 mm^3^ (IQR, 240.5–382.4 mm^3^) on the corresponding unaffected side (*p* < 0.001, Figure 4). The median ratio between DRG volumes of the affected versus unaffected side was 1.240 (IQR, 1.147–1.365), representing a median relative enlargement of +24.0% (IQR, +14.7% to +36.5%, Figure 4).

The subgroup of patients with L5 radiculopathy (*n* = 19) showed a median affected DRG volume of 323.7 mm^3^ (IQR, 263.7–377.5 mm^3^) versus 258.0 mm^3^ (IQR, 232.6–310.4 mm^3^) contralaterally (*p* < 0.001). The median ratio between the affected and unaffected DRG volume was 1.230 (IQR, 1.112–1.380) in this subgroup, representing a median enlargement of +23.0% (IQR, +11.2% to +38.0%).

The subgroup of patients with S1 radiculopathy (*n* = 9) demonstrated a median affected DRG volume of 548.5 mm^3^ (IQR, 408.0–572.6 mm^3^) versus 416.5 mm^3^ (IQR, 371.4–427.7 mm^3^) contralaterally (*p* = 0.004). The median ratio between the affected and unaffected DRG volume was 1.287 (IQR, 1.099–1.314) in this subgroup, representing a median enlargement of +28.7% (IQR, +9.9% to +31.4%).

DRG volume correlated with patient height on both the affected (Spearman ρ = 0.50, *p* = 0.007) and non-affected sides (ρ = 0.38, *p* = 0.047). Age showed a moderate negative correlation with non-affected DRG volume (ρ = −0.38, *p* = 0.047) and a trend toward negative correlation with affected DRG volume (ρ = −0.33, *p* = 0.08). Body weight and body mass index showed no relevant correlations with DRG volume (all |ρ| ≤ 0.28, all *p* ≥ 0.15).

## 4. Discussion

We demonstrate that lumbar DRGs show reproducible volumetric enlargement in symptomatic radiculopathy and that PCD-CT permits quantification of this pathological change on routine low-dose periinterventional imaging. The affected DRG showed a median 24.0% volume increase relative to the asymptomatic contralateral side (*p* < 0.001), with high inter-reader reliability (median DSC 0.78–0.85, median VS 0.93–0.95), confirming both hypotheses. While conventional CT lacks sufficient soft tissue resolution to visualize DRG morphology on procedural low-dose acquisitions, PCD-CT technology now enables reliable assessment of these structures and detection of inflammation-driven volumetric changes without additional examinations, radiation exposure, or MRI requirements.

Among the 40 patients who met all clinical inclusion criteria, 12 patients had to be excluded because adequate DRG delineation could not be achieved. Among the remaining 28 patients, the affected DRG was larger in 26; as the cohort was selected for therapeutic response, this proportion does not represent a diagnostic sensitivity.

The twelve exclusions had three causes: beam-hardening from spinal instrumentation, distorted foraminal anatomy in scoliosis, and insufficient signal-to-noise on the low-dose acquisition; representative examples are provided in Figure S1. DRG assessment on interventional acquisitions is inherently limited in patients with these constellations.

The observed DRG enlargement reflects established pathophysiological mechanisms underlying radicular pain. Mechanical nerve root compression triggers a local inflammatory cascade within the DRG, characterized by increased vascular permeability, protein extravasation, and edema formation [3,4]. This inflammatory response involves cytokine upregulation and immune cell recruitment, leading to nociceptor sensitization [3,4]. That DRG swelling correlates with pain severity [5] and responds to anti-inflammatory treatment [6] establishes it as a functionally relevant marker rather than an epiphenomenon. The ability to quantify this pathological DRG tissue response using PCD-CT provides a window into disease biology that conventional CT could not access.

Our findings align with the established MRI literature on DRG pathology in radiculopathy. Aota et al. demonstrated on MR myelography that compressed lumbar DRGs become visibly swollen, quantifying a 19% larger diameter on the affected side, with swelling severity correlating with pain intensity [5]. Tortora et al. detected a 22% DRG volume reduction paralleling a 43% pain decrease after radiofrequency treatment [6]. Our observed 24% volume increase is comparable in magnitude and confirms in a CT context what MRI has established. MR neurography studies have documented DRG enlargement across various neuropathic conditions [17,18], with normative data showing volumes ranging from 125 mm^3^ at L3 to 323 mm^3^ at S1 that scale with body height [19], consistent with our findings. Importantly, no systematic side-to-side differences exist in healthy individuals [19], confirming that the asymmetry we observed is most likely pathological.

Prior CT-based DRG assessment has been limited. Apostolidis et al. demonstrated that diagnostic CT scans with intravenous contrast in oncology patients can depict and measure DRG volumes [20], establishing feasibility on standard CT. However, their approach required dedicated soft tissue imaging with contrast enhancement for staging purposes. Radiculopathy patients do not typically receive such examinations, and these optimized protocols with contrast and soft tissue reconstruction are not present during interventional procedures. This highlights the gap our study addresses: demonstrating DRG assessment on low-dose interventional acquisitions without contrast enhancement.

The successful DRG visualization on low-dose interventional imaging reflects the technical evolution that PCD-CT represents over conventional systems. Direct photon counting eliminates electronic noise while enabling smaller detector elements without dose penalty, delivering substantially higher spatial resolution and contrast-to-noise ratio. Conventional CT cannot depict DRG morphology on low-dose acquisitions because the signal difference between DRGs and surrounding structures falls below the noise floor at interventional radiation doses. PCD-CT overcomes this limitation, making soft tissue neural structures accessible on intervention-grade protocols optimized for skeletal visualization. These advantages, demonstrated for vascular structures [9,10], temporal bone [11,12], and spinal anatomy [13], now prove sufficient to delineate small neural structures and detect biological tissue responses. PCD-CT enables sharper images with lower noise than conventional CT while reducing radiation dose by nearly half [13], repurposing incidental imaging from therapeutic injections for DRG assessment within standard clinical workflows.

Our findings have implications for understanding radiculopathy and advancing diagnostic imaging. Clinical localization of radiculopathy to a specific nerve root level demonstrates limited accuracy, particularly for sensory manifestations. Dermatomal sensory patterns show considerable overlap and individual variability, while myotomal deficits, though more reliable, also exhibit substantial inconsistency in determining the precise pathological level [21,22]. This diagnostic uncertainty is compounded when multiple spinal segments show degenerative changes, making objective morphological markers of nerve pathology particularly valuable. By detecting DRG changes on CT, we establish a biomarker bridging anatomical imaging and pathophysiology. Previous CT assessment was limited to indirect signs such as disc herniation or stenosis; direct visualization of the pathological tissue response provides more specific information about the nerve biological state, potentially improving diagnostic accuracy when multiple levels show degenerative changes. The ability to quantify DRG alterations on imaging already performed for therapeutic interventions creates opportunities for treatment monitoring, with serial volumetry potentially tracking conservative therapy response or predicting interventional treatment outcomes. From a research perspective, CT-based DRG assessment enables large-scale studies investigating relationships between DRG morphology, pain phenotypes, and treatment outcomes using routine clinical data. In the context of modern CT imaging technology, our findings exemplify how advances in detector physics translate to clinically meaningful capabilities. CT-guided puncture techniques have become and will remain a clinical standard for challenging spinal interventions [23]. By enabling visualization of biological processes in structures previously beyond CT’s reach, photon-counting technology transforms the diagnostic scope of the modality, with particular significance for interventional neuroradiology where integration of procedural and diagnostic information could streamline workflows.

Several limitations warrant consideration: The sample size (*n* = 28) was relatively small, and the retrospective design limits generalizability. The cohort was highly selected from 117 initially screened cases, including only patients with well-documented single-level radiculopathy responding to targeted PRT, which maximized the likelihood of detecting DRG changes but introduced selection bias. Clinical practice encompasses patients with ambiguous presentations or unclear therapeutic response. Because level assignment relied on response to level-specific PRT, the cohort carries a high pre-test probability of DRG pathology at the level examined. The proportion of 26 of 28 is therefore not a diagnostic sensitivity, and specificity cannot be assessed from the responder cohort alone. The findings are not transferable to unselected populations; DRG volumetry is not clinically validated and has no clinical consequence at present. This study is hypothesis-generating and addresses a pathophysiological surrogate marker rather than a diagnostic test. Our imaging protocol utilized routine intervention scans not optimized for soft tissue neural visualization (no contrast agent, standard bone kernel). Greater diagnostic yield might be achievable with dedicated parameters or spectral reconstructions. No MRI reference standard was available. Pre-interventional MRI was acquired for clinical indications without a uniform protocol, and clinical multislice sequences do not permit three-dimensional DRG segmentation.

Our approach requires PCD-CT technology, not yet universally available, though ongoing deployment will increase accessibility. While we established feasibility and detected measurable volumetric asymmetry, the clinical added value of routine DRG assessment during PRT requires prospective evaluation in larger cohorts with diverse presentations and outcome measures. Prospective validation should add standardized pain questionnaires before and after the intervention, which are not part of routine care in this setting, and high-resolution three-dimensional MRI at both time points. A control cohort undergoing comparable CT-guided injections not directed at the DRG, such as sacroiliac joint infiltration, would further strengthen inference.

## 5. Conclusions

Routine low-dose PCD-CT scans acquired during PRT can reliably depict and quantify volumetric enlargement of the affected DRG in selected radiculopathy patients. PCD-CT enables quantitative assessment of DRG pathology on procedural imaging, extracting biological information from therapeutic acquisitions. This extends CT-based neuroradiological research into biological domains previously accessible primarily through MRI and opens avenues for understanding radiculopathy mechanisms and monitoring treatment response.

## Figures and Tables

**Table 1:** Patient characteristics of the study cohort (*n* = 28).

Characteristic	Median (Interquartile Range) or Number (Percentage)
Age [years]	56.0 (48.8–63.2)
Female sex	15 (53.6%)
Male sex	13 (46.4%)
Height [m]	1.71 (1.65–1.80)
Weight [kg]	84.0 (73.5–96.2)
Body mass index [kg/m^2^]	27.7 (24.6–29.3)
Affected nerve root	
L5 (total)	19 (67.9%)
Left	10 (35.7%)
Right	9 (32.1%)
S1 (total)	9 (32.1%)
Left	5 (17.9%)
Right	4 (14.3%)

**Table 2:** Inter-reader comparison of DRG segmentations *.

Comparison	Dice Similarity Coefficient Median (Interquartile Range)	Volume Similarity Median (Interquartile Range)
Reader 1 vs. reader 2	0.85 (0.81–0.87)	0.95 (0.93–0.97)
Reader 2 vs. reader 3	0.78 (0.74–0.81)	0.93 (0.90–0.97)
Reader 1 vs. reader 3	0.78 (0.73–0.82)	0.94 (0.89–0.98)

* Values represent all 56 segmentation masks per reader (28 patients, two DRGs per patient). Values range between 0 and 1; values closer to 1 indicate better agreement. Abbreviations: DRG, dorsal root ganglion; IQR, interquartile range.

## Data Availability

The data used in this study have not been used in other studies. The datasets generated and analyzed during the current study are not publicly available due to data privacy reasons but are available from the corresponding author upon reasonable request.

## Supplementary Materials

The following supporting information can be downloaded at: https://www.mdpi.com/article/10.3390/diagnostics16213461/s1, Figure S1: representative cases in which dorsal root ganglion delineation was not possible; Table S1: dorsal root ganglion volumes in patients without therapeutic response to periradicular therapy.

## Abbreviations

The following abbreviations are used in this manuscript:

CTDIvol	Volume computed tomography dose index
DLP	Dose–length product
DRG	Dorsal root ganglion
DSC	Dice similarity coefficient
IQR	Interquartile range
kV	Kilovolt
L5	Fifth lumbar nerve root/dorsal root ganglion
mAs	Milliampere-second
mGy	Milligray
mm	Millimeter
ms	Millisecond
PCD-CT	Photon-counting detector computed tomography
PRT	Periradicular therapy
ρ	Spearman’s rank correlation coefficient
S1	First sacral nerve root/dorsal root ganglion
STAPLE	Simultaneous truth and performance level estimation
VS	Volume similarity
