# Supplementary material for: Photon-Counting Detector CT Enables Quantification of Dorsal Root Ganglion Enlargement in Radiculopathy
Source: Diagnostics (Basel). 2026 Oct 23;16(21):3461. doi: 10.3390/diagnostics16213461 (PMC13762250; doi:10.3390/diagnostics16213461)
Supplement: Supplementary file 1 [file diagnostics-16-03461-s001.zip › diagnostics-4421387-supplementary.pdf]

Supplementary Material

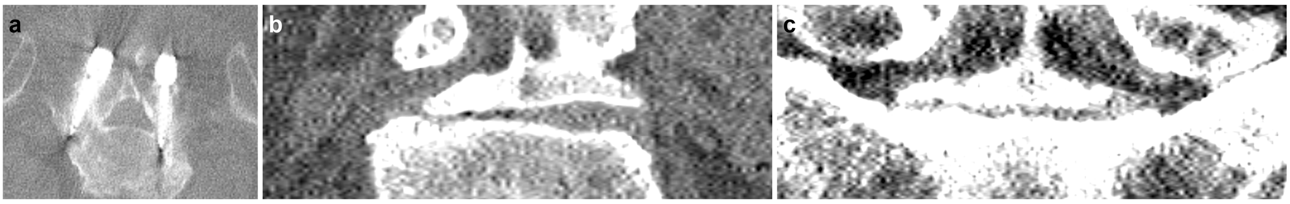

Figure S1. Representative cases in which dorsal root ganglion delineation was not possible.

Beam-hardening adjacent to spinal instrumentation, with hypodense streaks projecting into the neuroforamina (a); scoliotic deformity with rotated vertebral anatomy, so that the neuroforamina are not captured symmetrically in the reformation plane (b); and insufficient signal-to-noise ratio, with image noise exceeding the contrast between the dorsal root ganglion (DRG) and the surrounding tissue (c). In all three cases, the DRG could not be delineated with confidence on the low-dose planning acquisition, and the patients were excluded from the analysis. Abbreviations: DRG, dorsal root ganglion; PCD-CT, photon-counting detector computed tomography.

Table S1. Dorsal root ganglion volumes in patients without therapeutic response to periradicular therapy (n = 20).

ID	level	affected side	DRG volume (affected) [mm^3]	DRG volume (healthy) [mm^3]
1	L5	right	361.7	465.6
2	L5	left	493.5	417.8
3	L5	right	502.3	359.7
4	L5	right	260.4	261.9
5	L5	right	495.3	648.1
6	L5	right	417.8	385.4
7	L5	left	538.6	561.9
8	L5	left	187.3	211.5
9	L5	left	551.3	533.0
10	S1	right	619.4	667.0
11	S1	right	630.6	588.3
12	L5	left	502.8	418.4
13	L5	right	295.9	386.1
14	S1	right	629.9	562.5
15	L5	right	378.7	385.5
16	S1	left	627.5	639.2
17	S1	right	490.6	583.2
18	S1	left	662.9	535.9
19	L5	right	471.7	420.6
20	L5	left	439.8	391.4

Of 31 patients with documented absence of a therapeutic response following PRT, 20 had sufficient DRG delineation to allow retrospective evaluation as a separate cohort in a post hoc analysis. Segmentation was performed by the same three independent blinded readers on the same reformations, with STAPLE consensus and identical volumetric endpoints as in the main cohort. The subcohort comprised 14 patients with L5 and 6 with S1 radiculopathy. The median DRG volume was 494.4

mm^3 (IQR, 408.0–568.3) on the affected side and 443.1 mm^3 (IQR, 386.0–567.7) on the contralateral side; the paired Wilcoxon signed-rank test showed no statistically significant difference between the sides (P = 0.695). The median ratio between the affected and contralateral DRG volumes was 1.014 (IQR, 0.918–1.122), compared with 1.240 (IQR, 1.147–1.365) in the main cohort. In 10 of 20 cases (50.0%), the DRG volume was higher on the affected side, compared with 26 of 28 cases (92.9%) in the main cohort. Across the individual pairwise interrater comparisons, median Dice similarity coefficients ranged from 0.636 to 0.869, and median volume similarity values ranged from 0.847 to 0.955. Abbreviations: DRG, dorsal root ganglion; IQR, interquartile range; PRT, periradicular therapy.
